# Targeted inactivation and identification of targets of the Gli2a transcription factor in the zebrafish

**DOI:** 10.1242/bio.20136262

**Published:** 2013-09-09

**Authors:** Xingang Wang, Zhonghua Zhao, Julius Muller, Audrey Iyu, Alexis Jiaying Khng, Ernesto Guccione, Yijun Ruan, Philip W. Ingham

**Affiliations:** 1 Institute of Molecular and Cell Biology, 61 Biopolis Drive, Singapore 138673; 2 Genome Institute of Singapore, 60 Biopolis Street, Singapore 138672; 3 Department of Biological Sciences, National University of Singapore, 14 Science Drive 4, Singapore 117543; ‡ Present address: High Throughput Molecular Drug Discovery Center, Tianjin International Joint Academy of Biotechnology and Medicine, Tianjin, China; § Present address: The Jackson Laboratory, 600 Main Street, Bar Harbor, ME 04609, USA; ¶ Present address: Lee Kong Chian School of Medicine, 11 Mandalay Road, Singapore 308232

**Keywords:** Gli transcription factor, Target genes, Hedgehog signaling, ChIP-seq, Zebrafish, *gli2a* null allele

## Abstract

Hedgehog (Hh) signaling is mediated by the Gli transcription factors and, in the zebrafish, plays an important role in patterning both the neural tube and myotome. Using a null allele of the *gli2a* gene induced by targeted mutagenesis, we show that Gli2a is completely dispensable in the fish but acts redundantly with Gli1 to regulate expression of known Hh targets, such as *ptch2*, *prdm1a* and *eng2a*, in the myotome and neural tube. To identify novel targets of Hh signaling, we performed chromatin immunoprecipitation sequencing (ChIP-seq) of whole embryo extracts. Samples were significantly enriched for 192 genomic regions, some of which are associated with four known Hh target genes, *ptch1*, *ptch2*, *gli1* and *olig2*. Sequence analysis of these regions reveals a high level of conservation of Gli-binding sites from fish to mammals in some, but not all, cases. Expression analysis of other transcription units that are closely associated with peaks identified several putative targets not previously implicated as Hh targets, including *myl10*, *hnmt*, *lrp4*, *efemp2*, *fras1*, *quo*, and *lamc1*. Each of these genes shows loss of, or reduced expression in, embryos homozygous for an antimorphic allele of *gli2a*, *you-too* (*yot*), consistent with their being direct targets of Gli2a.

## Introduction

A major response of cells to Hedgehog (Hh) signaling is the upregulation of transcription of specific target genes. A classic example is provided by the gene encoding the Hh receptor Patched (*ptc/ptch*), upregulation of which in response to Hh signals creates a negative feedback loop that limits the range of the signal (Chen and Struhl, 1996). The role of Gli proteins as mediators of the transcriptional response of cells to Hh pathway activity is well established, but exactly how the different family members combine to achieve this is still only partially understood. The simplest scenario is found in *Drosophila*, where a single Gli protein, Cubitus Interruptus (Ci), is responsible for both the activation and repression of Hh target genes (Alexandre et al., 1996; Aza-Blanc et al., 1997; Méthot and Basler, 1999). Ci has both an N-terminal repressor domain and a C-terminal activator domain and is thus bi-functional, its activity being modulated by Hh-regulated proteolytic cleavage: in the absence of Hh, cleavage and proteolysis of the C-terminus yields a truncated form of the protein that can repress target gene expression. Activation of the Hh pathway abrogates this cleavage and primes the full-length form to activate transcription of target genes. In vertebrates, the situation is complicated by the presence of multiple Glis. The relative contributions of the Gli1, Gli2 and Gli3 proteins to the Hh response have been extensively analysed in mice (Bai and Joyner, 2001; Ding et al., 1998; Matise et al., 1998; Mo et al., 1997; Motoyama et al., 2003; Park et al., 2000). These studies have revealed that Gli1 is dispensable and functions exclusively as an activator to reinforce or amplify the response to Hh activity that is mediated by Gli2, which functions principally as an activator and Gli3, which functions principally as a repressor (Pan et al., 2006; Tyurina et al., 2005; Wang et al., 2000). Loss of function of either of these latter two Glis results in embryonic lethality with gross patterning defects, especially in the limbs and neural tube, that reflects their opposing roles in controlling Hh target gene expression. The picture that emerges from these *in vivo* functional analyses thus suggests a delicate balance of activator and repressor forms of both proteins underlies the differential response of cells to Hh signals (Stamataki et al., 2005).

Genome sequence analyses have revealed the presence of an additional Gli2 gene in several teleost species (including zebrafish, medaka, Tilapia, Fugu and Tetraodon) (supplementary material Fig. S1), introducing further complexity to the analysis of the Hh response in fish. Functional analysis of the teleost Gli genes has so far depended largely on transient knockdown studies using morpholino antisense oligonucleotide injection in the zebrafish embryo (Huang and Schier, 2009; Karlstrom et al., 2003; Ke et al., 2008; Tyurina et al., 2005; Wolff et al., 2003).These have revealed surprisingly subtle contributions of both the Gli2 proteins and Gli3. A loss of function mutation, *detour* (*dtr*), on the other hand, has demonstrated a critical requirement for the Gli1 protein in the zebrafish embryo (Chandrasekhar et al., 1999; Karlstrom et al., 2003), in contrast to its dispensable role in mammals. In addition, truncated repressor forms of the Gli2a protein encoded by the *you-too* (*yot*) mutant alleles of *gli2a* cause a phenotype equivalent to a complete loss of Hh signaling (Karlstrom et al., 1999; Varga et al., 2001), underling the critical role of Gli mediated transcriptional regulation for the Hh response.

Here we describe the generation and characterization of the first *gli2a* loss of function mutations in a teleost, which reveals the Gli2a protein to be dispensable both for normal embryonic development and survival to adulthood in the zebrafish. Despite this dispensability, double mutant analysis reveals that Gli2a acts as the principal mediator of the cellular response to Hh in the absence of Gli1. To identify genes regulated by Gli2a, we performed the first *in vivo* ChIP-seq analysis of an endogenous Gli protein. As well as confirming *ptch1*, *ptch2*, *gli1* and *olig2* as direct Hh targets, we identified a number of novel target genes that are induced by Hh signaling specifically in the mesoderm.

## Materials and Methods

### Zebrafish strains and husbandry

Adult fishes were maintained on a 14-hour light/10-hour dark cycle at 28°C in the AVA (Singapore) certificated IMCB Zebrafish Facility. Zebrafish strains used were *gli2a^ty119^* (Karlstrom et al., 1999), *gli1^ts269^* (Karlstrom et al., 1996), *smo^i640^* (Varga et al., 2001), *ptc1^hu1602^* and *ptc2^tj222^* (Koudijs et al., 2008).

### Generation, selection and genotyping of Gli2a mutant alleles

Plasmids encoding Zinc finger nuclease (ZFN) were synthesized by ToolGen, Korea. Capped mRNA was produced using mMessage mMachine kit according to the manufacturer's protocol and injected into 1-cell stage embryos with dosage of 50 pg per embryo. G0 adults derived from embryos injected with ZFN mRNA were in-crossed and their progenies were individually genotyped by PCR using the forward primer (5′-ATC AGC CAT ATT GGG CGA AAA A-3′) and the reverse primer (5′-GAG GGG TGT ACA CAT TTA TGC CAA GCA CT-3′) followed by Sanger sequencing using primer (5′-CTG GCT GGA CTC GGT GCT GGT GT-3′). *gli1^ts269^* mutants were genotyped by PCR using the forward primer (5′-CGA ATA TGG CAC AGG AGT GAT CTA TC-3′) the reverse primer (5′-TCC TCA CGC TGA TAC TGA CCT TGC-3′) and sequencing using the reverse PCR primer. The *gli2a^ty119^* mutants were genotyped by PCR using the forward primer (5′-GAG CCT TAA AAC TAG AAT GGC CA-3′) the reverse primer (5′-CCA TCA GTG GCC ATA TTT TCC-3′) and sequencing using the reverse PCR primer.

### Western blot analysis

Embryos were de-chorionated and de-yolked in ice-cold PBS without Ca^2+^ and Mg^2+^ in the presence of complete protease inhibitor cocktail (Roche). The embryo pellet was resuspended and lysed in RIPA buffer [50 mM Tris-HCl (pH 8.0), 150 mM NaCl, 1% NP-40, 0.5% sodium deoxycholate, 0.1% SDS, protease inhibitor cocktail and 1 mM PMSF], then centrifuged at 13,000 rpm for 10 minutes at 4°C. Supernatant was transferred to a new tube and the protein concentration was measured by Bradford assay using Bio-Rad Protein assay dye Reagent. 2× loading buffer (62.6 mM Tris-HCl pH 6.8, 2% SDS, 0.01% bromophenol blue, 10% glycerol and 100 mM DTT) was added to the supernatant and 20 µg total protein was loaded on each lane and separated on a 7.5% acrylamide denaturing gel at 30 mA for 120 minutes, and transferred onto Immobilon-P polyvinylidene fluoride (PVDF) membrane (Millipore). PVDF strips were blocked in 5% milk powder PBS 0.1% Tween20 for 1 hour, and incubated with rabbit anti-zebrafish Gli2a (1:5000) (Maurya et al., 2011) for 1 hour at room temperature. After washing, primary antibody was detected with ECL HRP-conjugated anti-rabbit IgG (1:50,000). Chemiluminescent Substrate was SuperSignal West Femto (Pierce). The loading amount of protein extract among specimens was evaluated by γ-tubulin level with mouse anti-γ-tubulin (1:5000, Sigma).

### Whole embryo ChIP-seq analysis

One thousand zebrafish embryos at either 5 and 15 somites stage (ss) were dechorionated by treating with pronase (1 mg/ml in embryo water), then de-yolked in cold PBS without Ca^2+^ and Mg^2+^ in presence of complete proteinase inhibitor (Roche). The embryonic cells were then fixed by 1% formaldehyde and chromatin immunoprecipitation (ChIP) was preformed following the protocol of Dahl and Collas using 5 µg of polyclonal anti-Gli2a antibody and 50 µl protein A beads (Invitrogen) (Dahl and Collas, 2008). The ChIP-seq library was constructed using the Illumina (USA) ChIP-seq DNA Sample Prep Kit following the manufacturer's instruction and sequenced by Solexa sequencing. MEME-ChIP (Machanick and Bailey, 2011) was used to detect significantly enriched motifs.

### *In situ* hybridization and immunofluorescence

All candidate genes were cloned into pGEM-T easy vector (Promega), and antisense probes were synthesized with Sp6/T7 Dig RNA labeling kit (Roche). Standard *in situ* hybridization and antibody staining was performed as previously described (Elworthy et al., 2008; Oxtoby and Jowett, 1993; Strähle et al., 1994). Primary antibodies were used at the following dilution: mAb 4D9 (anti-engrailed, DHSB) at 1:100, mAb F59 (anti-slow myosin heavy chain-1, DHSB) at 1:100, rabbit anti-Prox1 (Chemicon, USA) at 1:5000. The secondary antibodies were Alexa 488-conjugated goat anti-rabbit and Alexa 546-conjugated goat anti-mouse (1:1000, Invitrogen).

### Reverse transcription and quantitative PCR (Q-PCR)

Total RNA was isolated from embryos using Trizole (Invitrogen 15596-026) and reverse transcribed into cDNA with SuperScript™ III Reverse Transcriptase (Invitrogen 18080-093), following the manufacturer's protocols. Validation of selected ChIP-seq peaks was performed by Q-PCR of ChIP DNA form 5 ss and 15 ss embryos using specific primers (supplementary material Table S1).

Transcript levels of *smo*, *cdon* and *beta-actin 1*(*actb1*) were analysed by Q-PCR using specific primers (supplementary material Table S2).

## Results

### Generation of a null mutant allele Gli2a

**Fig. 1.**
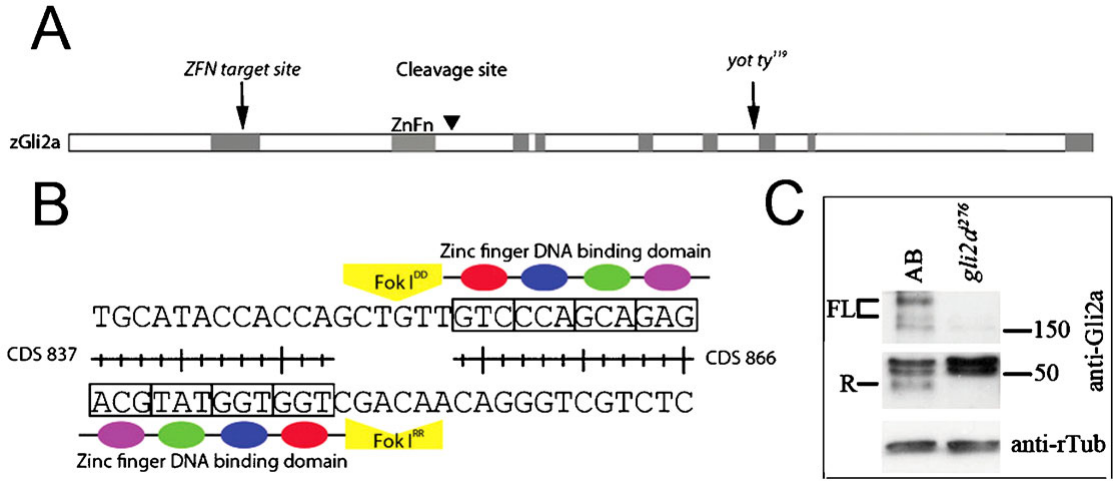
Targeted mutagenesis of zebrafish Gli2a. (A) Schematic structure of zebrafish Gli2a protein showing the zinc finger nuclease targeting site. Grey shading indicates regions of high conservation between species; ZnFn  =  Zinc finger DNA binding domain. (B) Nucleotide sequence of the zebrafish Gli2a gene selected for targeting. (C) Gli2a Western blot of 18 somite stage wild-type (AB) and *gli2a^i276^* homozygous whole embryo lysates. FL  =  full-length forms of Gli2a; R  =  truncated repressor form of Gli2a. γ-tubulin serves as a loading control.

To analyse the role of Gli2a in mediating the transcriptional response of cells to Hh, zinc finger nuclease (ZFN) mediated target mutagenesis was employed to generate a *gli2a* null allele. The *gli2a* ZFN was designed to target the fifth exon to generate mutants that should eliminate both Gli2a activator and repressor forms (Fig. 1A,B). Two new mutant alleles were recovered, one of which is associated with a 12-bp deletion while the other has a 4-bp insertion at the target site. These two mutants were designated *gli2a^i275^* and *gli2a^i276^*, respectively. Since the deletion in *gli2a^i275^* results in the loss of just four residues QLLS283-286, in a region that is not highly conserved between Gli proteins, we focused on the *gli2a^i276^* the insertion in which causes a frame shift resulting in a premature stop codon at CDS-1167 (supplementary material Fig. S2A). The resultant truncated open reading frame is predicted to encode a 388 aa protein, the first 284 residues of which correspond to the N-terminus of Gli2a. This includes the Gli2a repressor domain but lacks the zinc finger DNA binding domain and nuclear localization domain, both of which are essential for the transcription regulatory activity of Gli2a (Fig. 1; supplementary material Fig. S2H). Consistent with this, Western blot analysis of whole embryo lysates of *gli2a^i276^* homozygotes using the polyclonal antibody raised against residues 323–406 (Maurya et al., 2011) failed to detect either the 1439 aa full-length form or the truncated repressor form of Gli2a (Fig. 1C). The additional 104 amino acids encoded by the mutant allele have no homology to any known protein domain (data not shown); on this basis it seems unlikely that it would have any neomorphic or antimorphic activity. Consistent with this, animals homozygous for *gli2a^i276^* (and *gli2a^i275^*) are viable and show no morphological abnormalities either as embryos, larvae or adults (supplementary material Fig. S2B; Table S3).

### Redundant and overlapping requirements for Gli1 and Gli2a in the myotome and neural tube

**Fig. 2.**
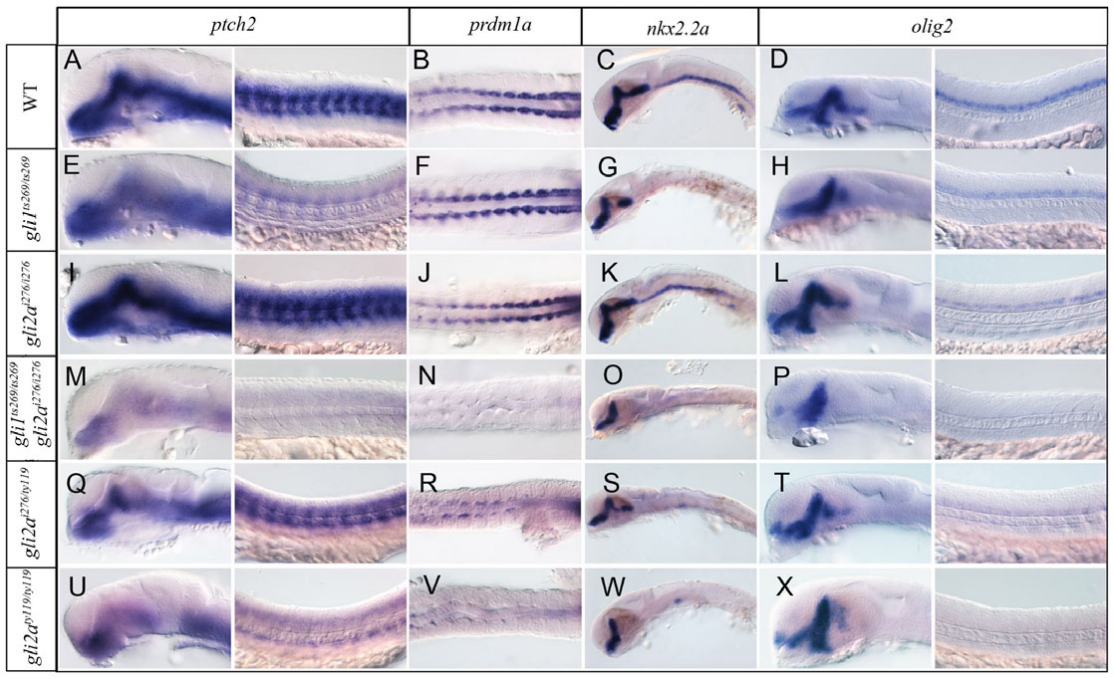
Expression of Hedgehog target genes in wild-type and *gli* mutant embryos. *ptch2*, *prdm1a*, *nkx2.2a* and *olig2* were examined in WT (A–D), *gli1^ts269/ts269^* (E–H), *gli2a^i276/i276^* (I–L), *gli1^ts269/ts269^*;*gli2a^i276/i276^* (M–P), *gli2a^i276/ty119^* (Q–T), and *gli2a^ty119/ty119^* (U–X) by WISH. All specimens were fixed at 24hpf, except those hybridized with *prdm1a* probe (18 somites).

**Fig. 3.**
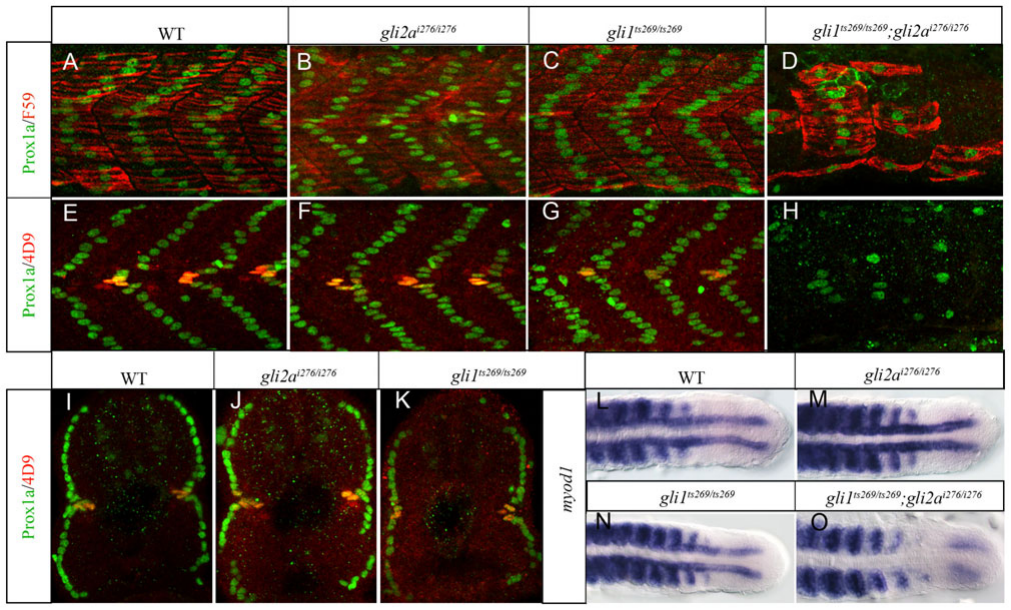
Gli1 and Gli2a act redundantly to pattern the zebrafish myotome. (A,E) Wild-type (WT); (B,F) *gli2a^i276^*; (C,G) *gli1^ts269^* and (D,H) *gli1^ts269^*; *gli2a^i276^* 24hpf embryos stained with anti Prox1a (green) and either mAb F59 (red: panels A–D) or 4D9 (red: panels E–H) to reveal slow-twitch muscle and MP fibres. (I–K) Transverse sections of embryos shown in panels E–G. (L–O) Dorsal views of the caudal regions of 18ss embryos hybridized with a probe for *myod1* transcript. (L) WT; (M) *gli2a^i276^*; (N) *gli1^ts269^* (O) *gli1^ts269^*;*gli2a^i276^*.

Consistent with the lack of morphological defects at 24 hpf, the expression patterns of *prdm1a*, which is normally activated in the adaxial cells in response to Hh signaling (Baxendale et al., 2004), and *nkx2.2a*, a target of Hh signaling in the ventral neural tube (Barth and Wilson, 1995), showed no change compared to wild-type embryos; the spatial patterns of two more Hh target genes *ptch2* (Concordet et al., 1996) and *olig2* (Stamataki et al., 2005) were also unaffected, though transcript levels appeared slightly increased or decreased, respectively (Fig. 2). Similarly, the domain of *nkx2.1b* expression in the anterior ventral telencephalon appeared unaffected (supplementary material Fig. S3) in contrast to the expansion previously reported to occur in *gli2a* morphant embryos (Huang and Schier, 2009; Karlstrom et al., 2003). Also in contrast to *gli2a* morphants (Wolff et al., 2003), *gli2a^i276^* homozygotes show normal specification and location of Engrailed (Eng)^+ve^ Muscle Pioneer (MP) cells and medial fast fibres (MFFs) (Fig. 3).

To confirm that Gli2a acts redundantly with Gli1 as previously inferred from morpholino based studies (Karlstrom et al., 2003; Wolff et al., 2003), *gli1^ts269^*; *gli2a^i276^* double mutants were generated. These displayed morphological defects similar to those of *smo^640i^* homozygotes (Varga et al., 2001), namely a curved trunk and u-shaped somites, a phenotype indicative of a failure to respond to Hh signaling (supplementary material Fig. S2E). Whole mount *in situ* hybridization (WISH) analysis revealed complete loss of *prdm1a* expression in adaxial cells and significant reduction of *ptch2* expression both in the somites and the CNS (Fig. 2). In addition, expression of both *nkx2.2a* and *olig2* was more severely downregulated than in *gli1^ts269^* homozygotes. Notably, the ventral patch of *nkx2.2a* expression in the midbrain was absent in the double mutant, as in *yot* homozygotes, indicating a specific requirement for Gli2a in this region of the brain (Fig. 2). Consistent with their loss of *prdm1a* expression, the *gli1^ts269^*; *gli2a^i276^* double mutants lacked nearly all slow-twitch muscle fibres, as revealed by the loss of Prox1a and slow myosin heavy chain 1 (Smyhc1) expression (Fig. 3). In addition, Eng expression in both MP and MFFs, was completely absent from the double mutants (Fig. 3). Taken together, these data confirm that Gli1 and Gli2a function redundantly in muscle specification and have partially overlapping roles in the neural tube.

### Gli2a is a more potent activator than Gli1 in the myotome

**Fig. 4.**
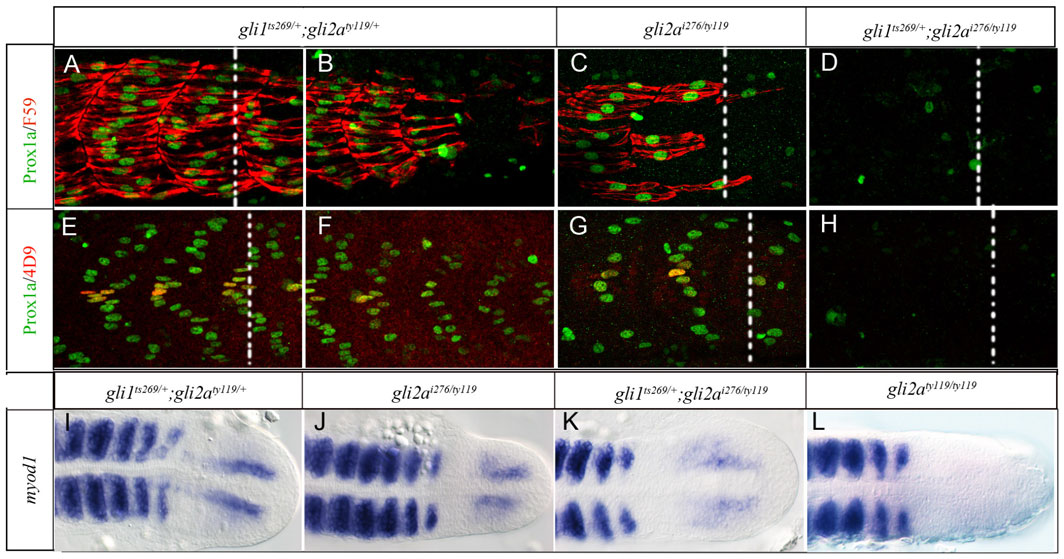
The Gli1 and Gli2a activators differ in their ability to counter Gli2a-mediated repression. (A,B,E,F) *gli1^ts269^*/+; *gli2a^ty119^*/+ (C,G) *gli2a^i276/ty119^* (D,H), and *gli1^ts269^*/+; *gli2a^i276/ty119^* (D) embryos at 24hpf stained with anti Prox1a (green) and either mAb F59 (red: panels A–D) or 4D9 (red: panels E–H) to reveal slow-twitch muscle and MP fibres. All panels show the caudal somites; the white dotted line indicates the position of the end of the yolk sac extension. The somites shown in panels C and G are located at 3–5 somites away from the end of the yolk sac extension. (I–L) Expression of *myod1* in (I) *gli2a^i276/ty119^*, (J) *gli1^ts269^*/+; *gli2a^ty119^*/+, (K) *gli1^ts269^*/+; *gli2a^i276/ty119^*, and (L) *gli2a^ty119/ty119^* embryos at 18 ss.

The Hh dependent processing of Gli2a (Ben et al., 2011) suggests that it should function both as an activator and repressor of Hh target genes. Animals homozygous for the *gli2a^ty119^* allele lack the activator form and express a truncated repressor form of Gli2a that effectively blocks the transcriptional response to Hh signaling in both the neural tube and somites resulting in a *smo* like phenotype (Karlstrom et al., 1999; Lewis et al., 1999b; Wolff et al., 2003). Trans heterozygous *gli2a^i276^/gli2a^ty119^* animals lack all activator activity but are expected to express the truncated repressor form at 50% of the level expressed in *gli2a^ty119^* homozygotes. Such embryos exhibited a morphological phenotype and effects on *ptch2, prdm1a, nkx2.2a* and *olig2* that are less severe than those of both *gli2a^ty119^* homozygotes and *gli1^ts269^; gli2a^i276^* double mutants (Fig. 2; supplementary material Fig. S2). Thus one copy of the Gli2a repressor cannot fully block target gene transcription in the presence of Gli1. Notably, however, there was significant variation in effects along the rostro-caudal axis of *gli2a^i276^/gli2a^ty119^* embryos. In the most rostral somites, the number of slow-twitch muscle fibres was only slightly reduced compared to wild type. By contrast, in the caudal somites, almost all slow fibres were lost. This regional difference was also reflected in the pattern of Eng expression: in rostral somites, the number of Eng^+ve^ cells was significantly decreased, but some cells remained. In more caudal somites, by contrast, all Eng^+ve^ cells were lost (Fig. 4A,E). To examine further the relative contributions of Gli1 and Gli2a in controlling target gene expression in the presence of the Gli2a repressor, we analysed slow fibres and MP specification in *gli1^ts269^*/+; *gli2a^ty119^*/+ and *gli1^ts269^*/+; *gli2a^i276^/gli2a^ty119^*. Although the *gli1^ts269^*/+; *gli2a^ty119^*/+ double heterozygote also showed loss of both slow fibres and MPs in caudal somites, the defects were weaker than in *gli2a^i276^/gli2a^ty119^*. In addition, *gli1^ts269^*/+; *gli2a^i276^/gli2a^ty119^* embryos, which have a single copy of the Gli1 activator but no full length Gli2a, lacked all slow fibres and MPs, a phenotype identical to that of *gli2a^ty119^* homozygotes (Fig. 4A–H). Consistently, although *myod1* expression was significantly reduced in embryos of all three genotypes, these effects appeared strongest in *gli1^ts269^*/+; *gli2a^i276^/gli2a^ty119^* embryos and weakest in *gli1^ts269^*/+; *gli2a^ty119^*/+ embryos (Fig. 4I–L). Taken together, these data indicate that Gli2a is more potent than Gli1 in overcoming the repressive activity of the truncated repressor form of Gli2a.

### Identification of direct targets of Gli2a by ChIP-seq analysis

To identify novel targets of Gli2a, ChIP-seq analysis of nuclear extracts from whole embryos at the 5 and 15 ss was performed using a rabbit anti-zebrafish Gli2a polyclonal antibody (Maurya et al., 2011) (see Materials and Methods for details). At least 9 million, 35 bp long reads per ChIP and input control sample were uniquely mapped by the CASAVA pipeline to the zv9 version of the zebrafish genome (Howe et al., 2013). MACS v2.09 was used for peak calling with default parameters and the average fragment length set to 150 bp (Zhang et al., 2008). A peak was labeled as significant if the corrected *P*-value was less than 0.01, the enrichment over input greater than 5 fold and the background corrected amount of reads contributing to a peak was more than 15. A sample of 17 putative Gli binding regions (GBRs) identified by peaks of varying strength were selected for validation by Q-PCR using specific primer sets together with primers for *tuba1*, *myh6* and *acat1* that served as negative controls (see Materials and Methods). 16/17 putative GBRs showed at least 2× enrichment in the 15 ss sample while enrichment of *hhip* was below background levels in both the 15 ss and the 5 ss samples (supplementary material Fig. S4). Moreover, the *tuba1* and *mhyc6* negative controls exceeded 2× enrichment in the 5 ss sample (supplementary material Fig. S4). Due to this enhanced background, a more stringent threshold of minimal reads contributing to a peak of 20 was applied. Using these parameters, we identified 93 peaks in the 5 ss sample and 122 peaks in the 15 ss sample, of which 23 are common to both samples (supplementary material Table S4). Notably, *nkx2.2a, pcdn, anapc* and *prdm8b* were excluded from both samples using these more stringent criteria, despite their being validated by the Q-PCR analysis.

The frequency of enrichment of the canonical Gli binding sequence (GBS), GACCACCCA or TGGGTGGTC (core sequence underlined) amongst the 15 ss peaks was significantly higher than amongst the 5 ss peaks (Table 1). Around 90% of all peaks detected are located within 100 kb of a transcription start site (TSS), with 29% of 5 ss peaks and 30% of 15 ss peaks located in close proximity (−5 to +2 kb) to a TSS. By contrast, Peterson et al. reported only 12% of GBRs detected by Gli1 ChIP seq analysis to be within 10 kb of a TSS (Peterson et al., 2012).

**Table 1.**
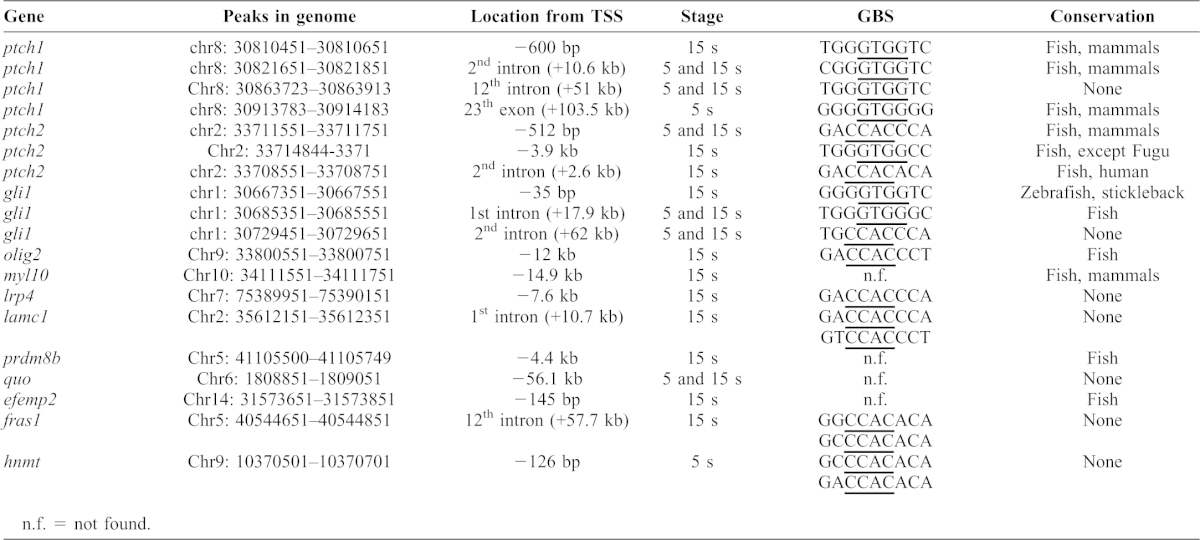
Genomic location of Gli2a bound region, predicted GBS and conservation between species.

### Sequences enriched in Gli2a ChIP are associated with known Hh target genes

Three genes encoding Hh pathway components that are also subject to regulation by Hh, namely *ptch1*, *ptch2* and *gli1* were associated with peaks, consistent with their previous identification as Hh target genes (Concordet et al., 1996; Karlstrom et al., 2003; Lewis et al., 1999a).

**Fig. 5.**
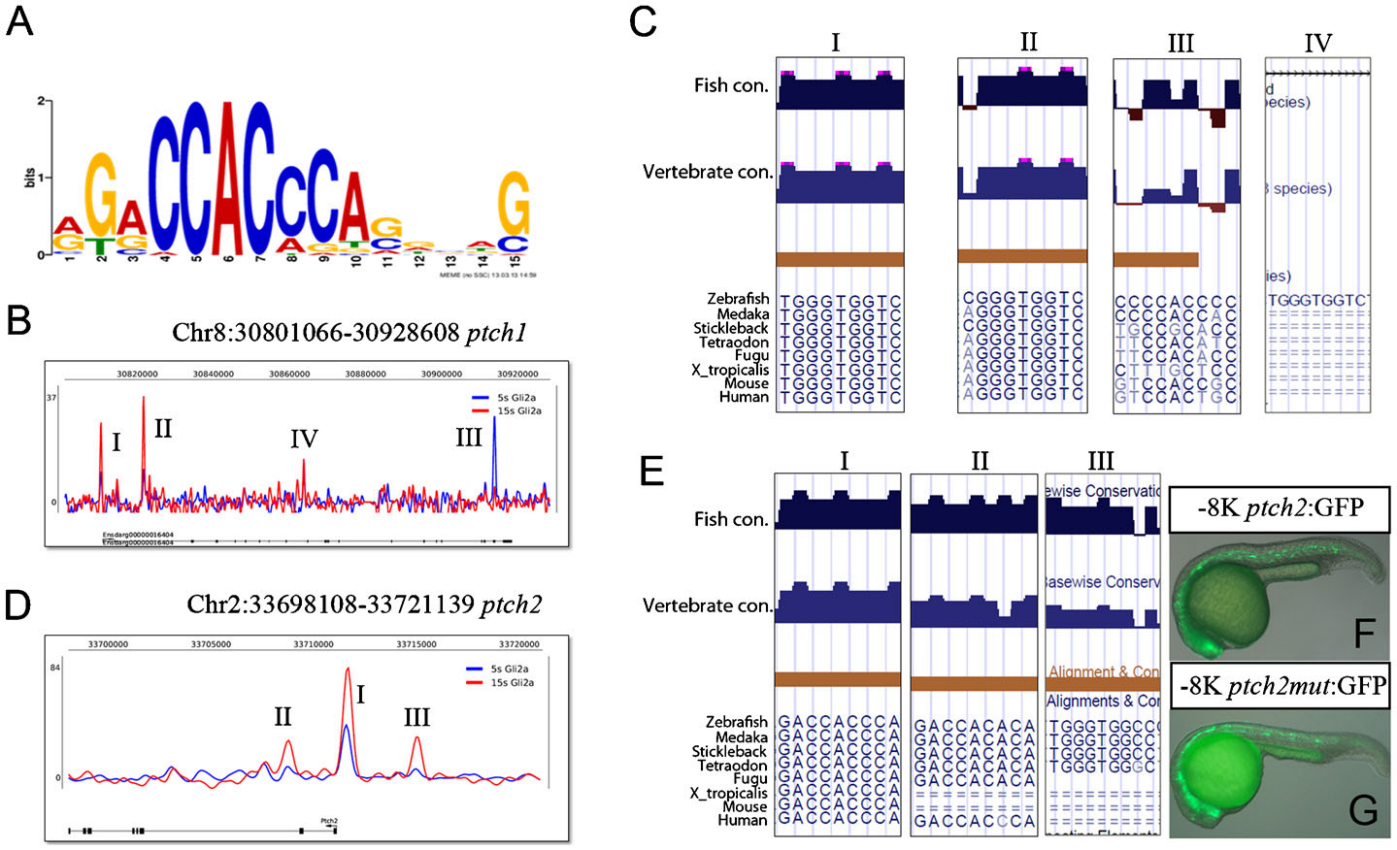
Gli bound region on *ptch1* and *ptch2* genomic locus. (A) The consensus Gli binding site (GBS). (B) Profile of Gli2a ChIP-seq sequence enrichment at the *ptch1* genomic locus. (C) Predicted GBS located within each peak (I–IV) and their conservation between different species. (D) Profile of Gli2a ChIP-seq sequence enrichment at the *ptch2* genomic locus. (E) Predicted GBS located within each peak (I–III) and their conservation between different species. (F) Transient expression of GFP driven by a wild-type −8 kb *ptch2* promoter fragment that includes the Peak I GBS. (G) Transient expression of the same reporter in which the GBS is mutated.

There were four distinct peaks at the *ptch1* locus: at −600 bp (I), and in intron 2 (II), exon 23 (III) and intron 12 (IV) (Fig. 5B). Peaks II and IV were detected both at 5 ss and 15 ss, while peaks I and III were detected only at 15 ss and 5 ss, respectively, implying that *ptch1* regulation at different stages may be dependent on specific enhancers. Peak I contains a single canonical GBS that is highly conserved between multiple fish species and mammals, including human. Previous studies have shown that this site mediates the Hh responsiveness of a human PTCH1 reporter gene in tissue culture cells (Ågren et al., 2004). Peak II, the mostly highly enriched at 15 ss, contains a near perfect GBS (CGGGTGGTC) that is also conserved across fish species and in human. Peak III region (20^th^ exon) is relatively conserved among all species, containing a degenerate GBS (GGGGTGGGG) (Fig. 5C). Peak IV, detected both at 5 ss and 15 ss, contains a perfect GBS (TGGGTGGTC) but is not conserved in any other species for which sequence data are available.

The *ptch2* locus is associated with three distinct peaks, each of which was detected at 15 ss but only one (Peak I) at 5 ss; this is located at −550 bp in a region that is highly conserved between fish species and mammals and includes a perfect GBS (Fig. 5D,E). Peak II, which is located in intron 2, contains a near perfect GBS (GACCACACA) that is conserved across fish species and also in human, though not in mouse or *Xenopus* (Fig. 5D,E). Peak III, located 3.9 kb upstream of the TSS, contains a prefect GBS (TGGGTGGCC) that is conserved in all fish species for which data are available, except *Fugu*, but not in mammals. An 8 kb fragment upstream of the *ptch2* TSS that includes the region corresponding to Peak I is sufficient to drive reporter gene expression in a pattern similar to that of the endogenous *ptch2* gene (A. K. Maurya and P.W.I., unpublished; Fig. 5F). Mutation of the Peak I GBS in this reporter construct had no obvious effect on its expression in transient transgenic embryos (Fig. 5G).

**Fig. 6.**
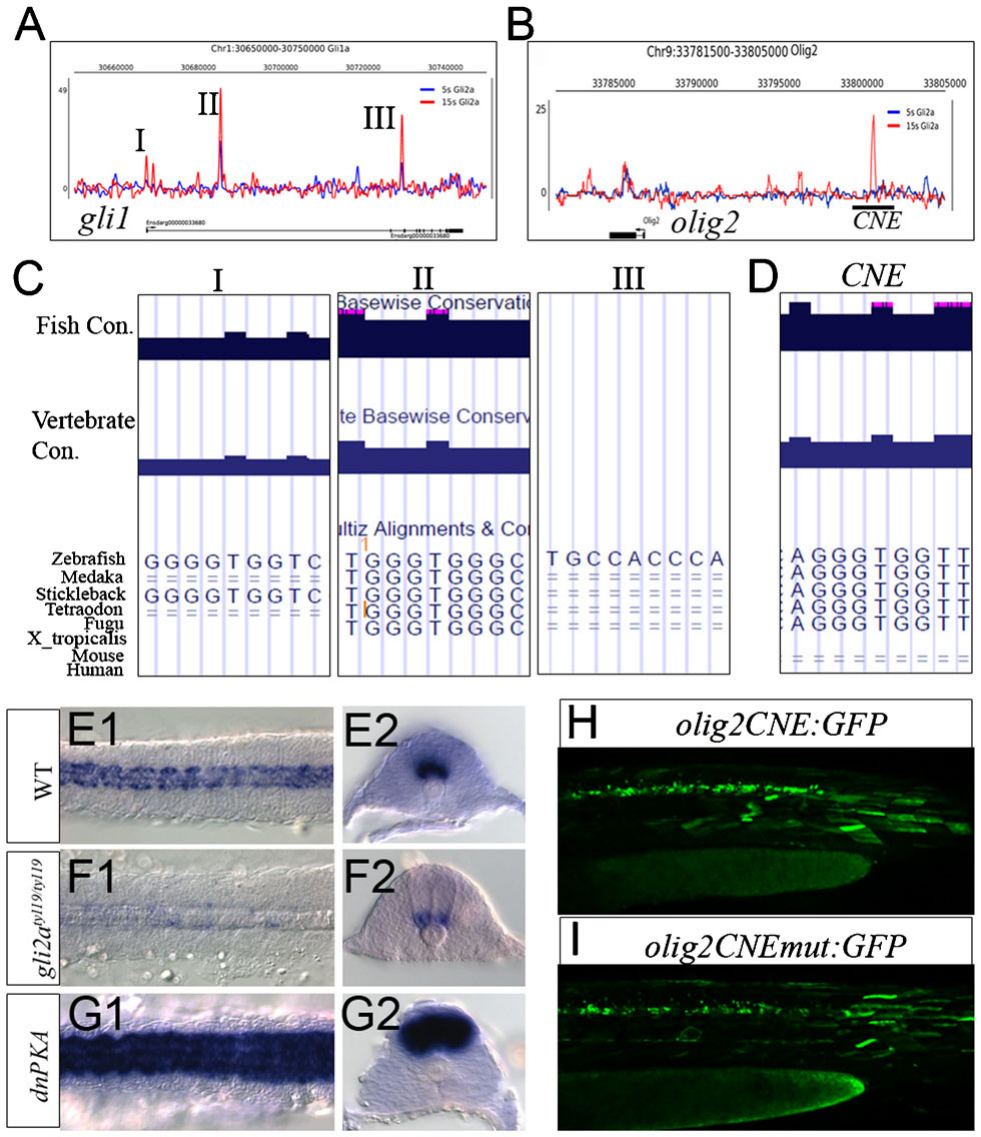
Gli bound region on *gli1* and *olig2* genomic locus. (A) Profile of Gli2a-ChIP sequence enrichment at the *gli1* genomic locus. (B) Profile of Gli2a-ChIP sequence enrichment at the *olig2* genomic locus. (C) Predicted GBS located within each *gli1* peak (I–III) and their conservation between different fish species. (D) Predicted GBS located within the *olig2* peak (CNE) and its conservation between different fish species. (E–G) Expression of *olig2* transcript in wild-type (E1,E2), *gli2a^ty119^* (F1,F2) and dnPKA injected (G1,G2) embryos at the 15 somite stage. Left panels are dorsal view of mesoderm and right panels are section view. (H) Transient expression of GFP driven by a 1.9 kb fragment including the CNE (I) transient GFP expression driven by the same 1.9 kb fragment in which the GBS was mutated.

At the *gli1* locus, one peak was detected close to the TSS only in 15 ss embryos, while two peaks (II and III) in intronic regions were detected at both 5 ss and 15 ss, though with higher levels of enrichment at 15 ss (Fig. 6A). Peak I includes a near perfect GBS (GGGGTGGTC) located at −88 bp to −73 bp, that is absolutely conserved in the stickleback while Peak II, located in the 1^st^ intron, contains a near perfect GBS (TGGGTGGGC) that is conserved across all five fish species examined, but is not present in amniotes. Peak III is located in 2^nd^ intron with a near perfect GBS (TGCCACCCA), but it is not conserved with any species (Fig. 6C).

Interestingly, transcription units encoding another two Hh signaling components Cdon and Smo were also found to be associated with Gli2a ChIP peaks detected at both 5 ss and 15 ss (supplementary material Fig. S5A,B). The peak close to *smo* located at −1 kb from the TSS contains a near perfect GBS (TGTGTGGTC) that is not conserved between species. The single peak 50 bp upstream of the *cdon* TSS detected at both 5 ss and 15 ss lacks a GBS. Transcript levels of *smo* and *cdon* were analysed by Q-PCR in wild-type embryos and in embryos in which the response to Hh is blocked (*gli2a^ty119^* homozygotes) or constitutively activated (by injection of mRNA encoding a dominant negative mutation of the PKA regulatory subunit (dnPKA)). Final relative expression levels of *smo* and *cdon* were normalized to the levels of *actb1* transcript. We found that *cdon* expression was upregulated in *gli2a^ty119^* mutant embryos and repressed in embryos injected with dnPKA mRNA; this is consistent with the previous finding that *Cdon* is repressed by Shh signaling in mouse embryos (Tenzen et al., 2006). *smo* transcript levels appeared unchanged in both genotypes (supplementary material Fig. S5).

Expression of *olig2*, a marker gene of motor neuron differentiation, is also known to be dependent upon Hh signaling in mammals and zebrafish (Lu et al., 2000; Stamataki et al., 2005). Consistent with this, *olig2* is overexpressed by ectopic activation of Hh (dnPKA injected), while its expression is largely reduced in *gli2a^ty119^* homozygous embryos (Fig. 6E–G). Previous studies in mouse have identified a 2.2 kb fragment containing 3 GBS upstream of the *olig2* promoter that can recapitulate the endogenous expression pattern in the neural tube (Wang et al., 2011). A single peaks of enrichment associated with the *olig2* locus at −12 kb, was detected exclusively at 15 ss (Fig. 6B). This peak includes a single degenerate GBS (AGGGTGGTT) that is absolutely conserved in all five fish species examined, but is not found in tetrapods and mammals (Fig. 6D). To investigate the functional significance of this peak, a 1.9 kb fragment containing a relatively conserved non-coding region (CNE) was inserted upstream of a *β-globin* minimal promoter driving GFP to test for enhancer function. This construct recapitulated the expression of *olig2* in the neural tube in transient transgenic embryos (Fig. 6H). Mutation of the GBS within the CNE, however, caused only a slight reduction in level of transgene expression in the neural tube (Fig. 6I).

### Novel Hh target genes identified by Gli2aChIP

**Fig. 7.**
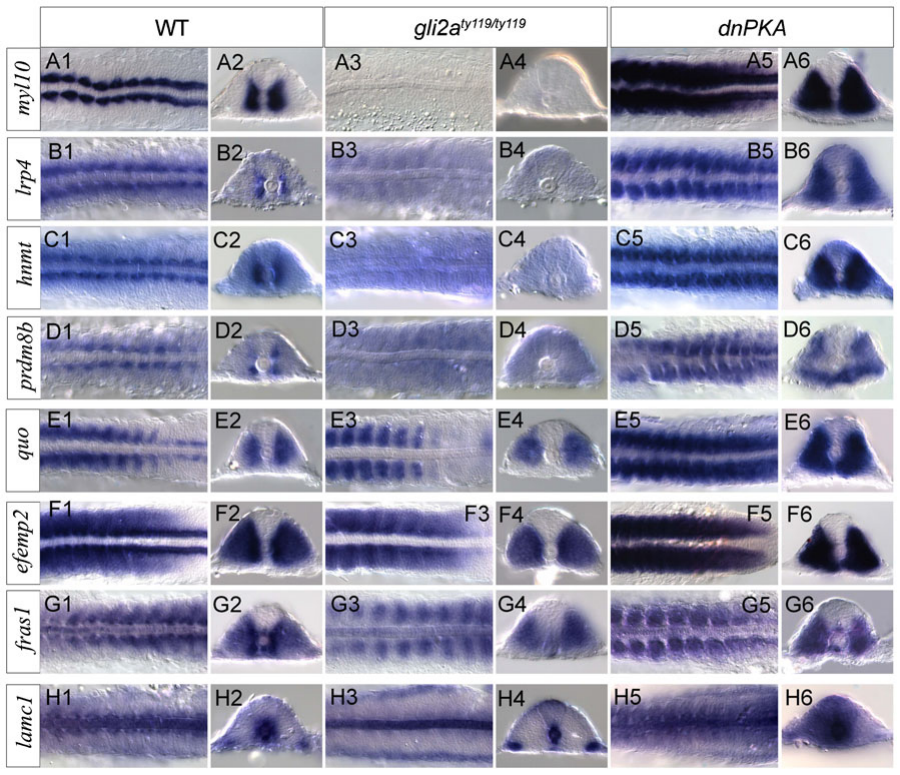
Validation of new Gli2a targets by WISH. (A–H) Expression of seven newly identified Gli2a direct targets plus *prdm8b* was analysed by WISH in wild-type, *gli2a^ty119^* and dnPKA injected embryos at the 15 somite stage. Each pair of panels shows a dorsal view of mesoderm of a flat mounted embryo and a transverse section of a similar stage embryo.

To identify novel Hh target genes, we cloned 37 genes associated with Gli2a ChIP peaks and analysed their patterns of expression in embryos by WISH. To limit the sample size, we selected only genes containing a Gli2a peak or located immediately downstream of it, but also included *prdm8b*, based on its recent identification as a target of Gli1 (Peterson et al., 2012). Amongst this sample, 26 genes showed spatially restricted patterns of expression (supplementary material Table S5). To determine if these patterns are Hh dependent, we analysed expression of each gene in dnPKA-injected embryos and in *gli2a^ty119^* homozygous embryos. Eight genes were found to have altered expression patterns in these embryos; in each case, the gene was normally expressed in the adaxial cells and this expression was diminished or abolished in *gli2a^ty119^* embryos whilst in dnPKA-injected embryos, it expanded into the paraxial mesoderm (Fig. 7). Of these 8 genes – *myl10*, *lrp4*, *hnmt*, *quo*, *prdm8b*, *efemp2*, *fras1* and *lamc1* – 5 are associated with a GBR containing a GBS though none has previously been identified as a target of Hh signaling. Laminin C1 (Laminin γ1), the product of *lamc1*, has, however, been implicated in controlling the specification of the Hh dependent MP cells (Dolez et al., 2011). Both the *lamc1* and *fras1* genes are also expressed in the neural tube, the expression of the latter being restricted to the ventral floor of the neural tube. While this expression was reduced in *gli2a^ty119^* embryos, there was no evidence of its expansion in dnPKA-injected embryos. Neural tube expression of *lamc1*, by contrast, appeared to be upregulated in dnPKA-injected embryos.

**Fig. 8.**
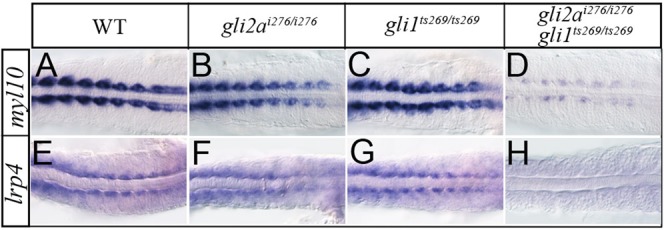
New Gli2a targets are redundantly controlled by Gli1 and Gli2a. Expression of *myl10* and *lrp4* expression in wild-type (A,B), *gli2a^i276^* (C,D), *gli1^ts269^* (E,F), and *gli1^ts269^*; *gli2a^i276^* (G,H) 18 somite stage embryos.

To investigate the functional redundancy of Gli1 and Gli2a further, we analysed expression of two of the newly identified target genes, *myl10* and *lrp4*, in *gli1* and *gli2a* single mutants and *gli1; gli2a* double mutants. Adaxial expression of both genes was largely unaffected in single mutant embryos but almost eliminated in double mutants (Fig. 8).

## Discussion

Prior to this study, the only *gli2a* mutants identified in the zebrafish were the *yot* alleles, both of which encode truncated forms of the protein that are predicted to act as constitutive transcriptional repressors (Karlstrom et al., 1999). Consistent with this, embryos homozygous for *yot* alleles exhibit a phenotype very similar to that caused by a complete loss of response to Hh signaling, as exemplified by homozygous *smo* mutants (Varga et al., 2001). Accordingly, analysis of Gli2a function has thus far been limited to the effects of morpholino antisense oligonucleotide-mediated knockdown of Gli2a expression. Remarkably, morpholinos targeting the *gli2a* transcript are able to suppress the *yot* phenotype fully, not only demonstrating their efficacy but also implying that Gli2a function is largely dispensable in the zebrafish (Karlstrom et al., 2003; Wolff et al., 2003). Thus in the myotome of morphants, all Hh cell types appear to be specified, with only a subtle effect on the positioning of MP cells noted (Wolff et al., 2003). One limitation of the morpholino approach is the transient nature of knockdown, precluding analysis of gene function beyond the first 4–5 days of development. In addition the difficulty in achieving 100% knockdown together with the well documented potential for off target effects, mean that conclusions based on this strategy must always be treated with caution. By generating null alleles of *gli2a* we have circumvented these limitations and have been able to analyse the effects of complete loss of Gli2a protein at all stages. While our findings broadly support the conclusions drawn from the previous studies, they go beyond them in establishing that Gli2a is completely dispensable for normal embryogenesis; we saw no effect on any Hh dependent cell types, including location of the MP cells. Moreover, we have found that Gli2a is also dispensable throughout larval and adult stages. These findings stand in remarkable contrast to the situation in mammals, where there is an absolute requirement for Gli2 function during embryogenesis. Of course, it is possible that the presence of the Gli2b protein in teleost genomes can compensate for the loss of Gli2a function. However, we find this unlikely for two reasons: first, previous reports of *gli2a;gli2b* double morphants described only relatively minor phenotypic effects of the knockdown of both proteins; and second, our demonstration that *gli1;gli2a* double mutants exhibit a strong loss of Hh response, which implies that Gli2b is unable to compensate for loss of Gli2a. This latter finding implies that in the mesoderm at least, the response of cells to Hh signals is mediated principally by Gli1 and Gli2a. This interchangeability between Gli1 and Gli2a is striking given the differences between the two proteins; whereas Gli2a contains functional activator and repressor domains, Gli1 is exclusively a transcriptional activator (Dai et al., 1999). Based on the characteristics of the mammalian Gli2 protein, this may not seem so surprising; in contrast to Gli3, Gli2 is inefficiently processed to its repressor form and in line with this, mutant analyses have shown that Gli2 also functions principally as an activator (Ding et al., 1998; Matise et al., 1998). Moreover, targeted replacement has shown that Gli1 can substitute for Gli2 when expressed under the control of the endogenous Gli2 regulatory elements (Bai and Joyner, 2001). By contrast to mammalian Gli2, however, zebrafish Gli2a is efficiently processed to a truncated putative repressor form in the absence of Hh input (Ben et al., 2011). Despite this, our analysis provided little evidence of a repressor function for Gli2a; embryos homozygous for the null allele showed no evidence either of an upregulation Hh target genes such as *ptch2*, or of the ectopic expression of Hh target genes, such as *nkx2.1b*. Rather, the principal function of Gli2a, revealed in the absence of Gli1, is the activation of target genes, both in the myotome and neural tube. Interestingly, although Gli1 and Gli2a must regulate the same set of target genes, our analysis of *gli2a^ty119^* hemizygotes suggest that Gli2a is more effective than Gli1 in countering the repressive activity of the mutant truncated form of Gli2a, since *gli1^ts269^/+; gli2a^ty119^/+* double heterozygotes show a weaker phenotype than *gli2a^i276^* hemizygotes.

To identify Gli2a targets we used ChIP-seq analysis, an approach previously employed in mouse to identify targets of Gli1 and Gli3 (Lee et al., 2010; Peterson et al., 2012; Vokes et al., 2007; Vokes et al., 2008). In contrast to these studies, which relied upon the use of epitope tagged forms of the Gli proteins, we used a polyclonal antibody to pull down chromatin associated with the endogenous Gli2a protein. We analysed chromatin extracted from two different embryonic stages, with the expectation that this would allow us not only to validate peaks but also identify stage specific binding patterns. As expected, ChIPed samples from both stages showed significant enrichment for multiple sequences compared to input controls; however, the number of peaks detected was lower than reported for previous Gli1 and Gli3 ChIP analyses in mouse. The number of peaks can be increased by lowering the parameter score but this is accompanied by a decrease in the proportion of peaks containing the canonical GBS. Previous analyses have indicated that as many as 45% of sequences associated with Gli proteins lack recognizable GBS (Vokes et al., 2008) and it seems plausible that at least some of these represent *bona fide* targets; indeed, the peaks associated with 4/8 of the novel targets that we validated by WISH lack a recognizable GBS. Nevertheless, we selected to restrict our analysis to peaks detected using a parameter score of 20. Although comparable numbers of peaks were detected at 5 ss and 15 ss, the proportion of these containing GBS was considerably higher at 15 ss.

As anticipated, peaks associated with known Hh target genes, *ptch1, ptch2, gli1* and *olig2* were identified in both samples; however, no peaks were detected in close proximity to *hhip* another known direct target. In mouse, the 1^st^ intron of *hhip* contains a canonical GBS that binds both Gli1 and Gli3 (Vokes et al., 2007; Vokes et al., 2008); although this sequence is conserved in the mouse and fish genes, it is neither enriched in our ChIP-seq nor positive in Q-PCR assays, indicating that *hhip* may be targeted specifically by Gli1. In addition, a distantly located peak containing several GBS was identified by Gli1 at ChiP −256 kb in mouse, a region also not conserved in zebrafish. We did, however, find a single peak at −92 kb but only in the 5 ss sample, containing a perfect GBS GACCACCCA which is conserved among fish except *Tetraodon* and could be required for *hhip* regulation. Equally surprising was the failure to identify peaks associated with the *prdm1a* and *myod1*, both of which are regulated by Hh signaling in the adaxial cells of zebrafish embryos (Concordet et al., 1996; Baxendale et al., 2004). Moreover, *myod1* has recently been reported to be a direct target of Gli2 in mammalian cells (Voronova et al., 2013) and a GBR has been identified downstream of the *prdm1* gene in mouse and shown to act as a Shh responsive limb specific enhancer (Vokes et al., 2008).

The *ptch1* and *ptch2* genes exhibit a notable difference in their response to Hh signaling between mammals and teleosts; in mouse, *ptch1* is expressed at high levels in cells in close proximity to sources of Hh proteins and is highly upregulated in response to ectopic signaling activity, while *ptch2* is expressed at much lower levels adjacent to and in some cases co-expressed with Hh expressing cells (Motoyama et al., 1998). In this regard, it is notable that in mouse, the *ptch1* gene is located in the most GBS-rich region of the genome and itself harbours 14 GBS-containing Gli binding regions (GBRs), whilst *ptch2* harbors only three such regions (Peterson et al., 2012). In fish and avians, the sensitivity of the *ptch* genes to Hh signaling is reversed, with *ptch2* showing a much stronger response to Hh signals than *ptch1* (Concordet et al., 1996; Lewis et al., 1999a; Pearse et al., 2001). In line with this, only 4 of the 14 *ptch1* GBRs found in mouse are conserved in fish and of these, only two were detected in our ChIP analysis (supplementary material Table S6). However, we also detected only three peaks of Gli2a binding associated with *ptch2* in zebrafish, just one of which is conserved in mouse. Detailed dissection of the promoters of both genes will be required to elucidate the basis of their differential response to Hh signaling.

In the case of the *gli1* and *olig2* genes we identified peaks associated with GBS in similar locations to those found in the mammalian orthologues of these genes, which, however, are not conserved between fish and mammals. The conservation of *ptch* promoters could suggest that it is important to ensure the presence of negative feedback for Hh signaling, misregulation of which would cause cancer. Though positive feedback through *gli1* may be important to enhance Hh in some situation to promote cell differentiation and development, it certainly increases the risk of Hh misregulation.

To identify novel *bona fide* Gli2a target genes, we focused on those genes that are in close proximity to peaks (within 5 kb of their TSS). Of the 37 genes that we analysed, we found seven novel targets that have spatially regulated patterns of expression that are controlled by Hh signaling. Notably, all of these are expressed in the mesoderm, with only one, *lamc1*, showing evidence of Hh regulated neural tube expression. None of these has previously been implicated in the response to Hh signaling; they encode a heterogeneous set of proteins, including three extra cellular matrix proteins: EGF-containing fibulin-like extracellular matrix protein 2 (*efemp2*), Fraser syndrome causing protein 1(*fras1*) and lamininγ1 (*lamc1*). Interestingly, embryos mutant for lamininγ1 fail to differentiate Hh-dependent MP cells in the myotome, an effect that has previously been attributed to a role for attenuating the inhibitory effects of BMP signals. The finding that the *lamc1* gene is a Gli2a target suggests a possible link between Hh signaling and BMP regulation that could impact on MP specification, a possibility that is currently under investigation.

## Supplementary Material

Supplementary Material
