# Supplementary material for: Targeted inactivation and identification of targets of the Gli2a transcription factor in the zebrafish
Source: Biol Open. 2013 Sep 9;2(11):1203–13. doi: 10.1242/bio.20136262 (PMC3828767; doi:10.1242/bio.20136262)
Supplement: Supplementary Material [file supp_2_11_1203__index.html]

Targeted inactivation and identification of targets of the Gli2a transcription factor in the zebrafish — Supplementary Material 

# Targeted inactivation and identification of targets of the Gli2a transcription factor in the zebrafish

## bio.20136262 Supplementary Material

**Files in this Data Supplement:**

- Supplementary Material - Xingang Wang et al. doi: 10.1242/bio.20136262
- Table S4 - Genomic sequences detected by Gli2a ChIP-seq analysis.
