# Supplementary material for: Targeted inactivation and identification of targets of the Gli2a transcription factor in the zebrafish
Source: Biol Open. 2013 Sep 9;2(11):1203–13. doi: 10.1242/bio.20136262 (PMC3828767; doi:10.1242/bio.20136262)
Supplement: Supplementary Material [file supp_bio.20136262_bio.20136262-s1.pdf]

Biology Open

Supplementary Material

Xingang Wang et al. doi: 10.1242/bio.20136262

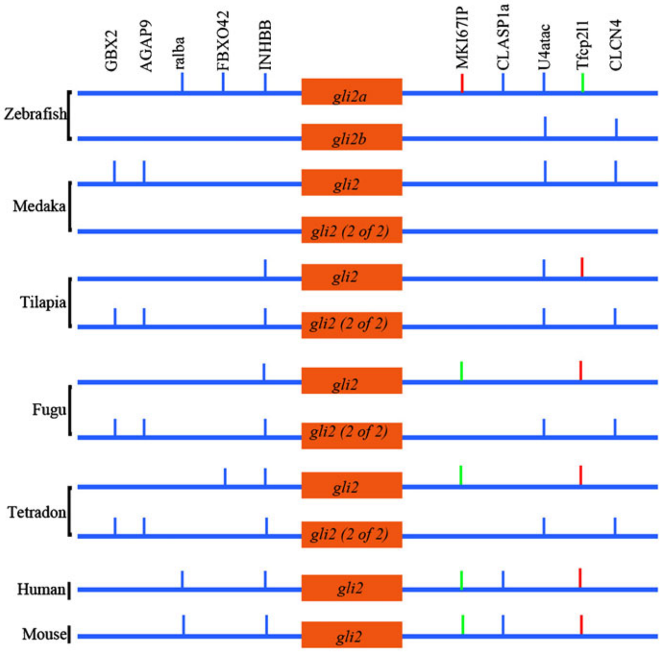

Fig. S1. Synteny relationships of teleost and mammalian *Gli2* genes.

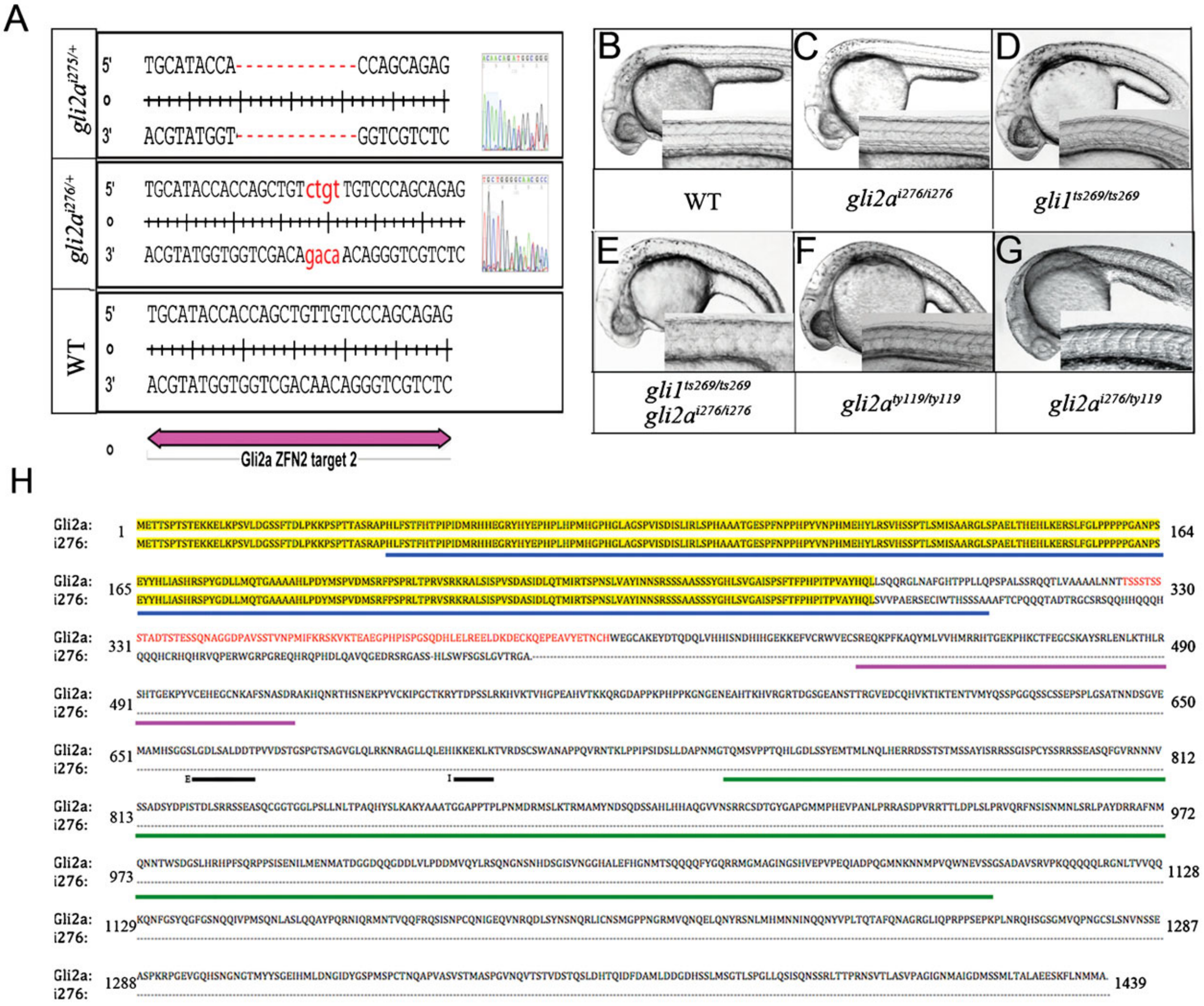

Fig. S2. Sequence information and morphological phenotype of new gli2a mutant alleles. (A) Sequence information of *gli2a*$^{i275/+}$and *gli2a*$^{i276/+}$at ZFN target site. (B–G) Morphological phenotype of wild type, *gli2a*$^{i276/i276}$, *gli1*$^{ts269/ts269}$, *gli1*$^{ts269/ts269}$;*gli2a*$^{i276/i276}$, *gli2a*$^{ty119/ty119}$, and *gli2a*$^{i276/ty119}$ at 30 hpf. (H) Alignment of Gli2a and Gli2a^{i276} amino acid sequence. Sequences highlighted in yellow show the common amino acids between them, sequence in red indicates the antigen region for generating the Gli2a antibody used in this study. Sequences underlined indicate the Gli2a repressor domain (blue), zinc finger DNA binding domain (pink), nuclear localization sequences (black) and activator domain (green). All domains were annotated by alignment with the human GLI2 protein (Fernández-Zapico, 2008).

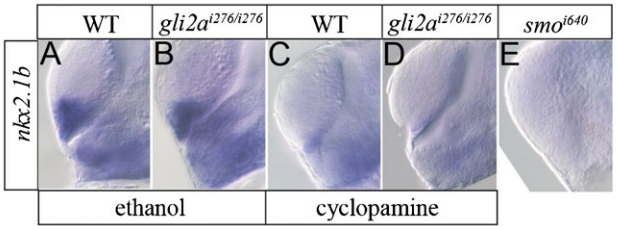

Fig. S3. *nkx2.1b* expression is not changed in *gli2a*$^{i276/i276}$. *nkx2.1b* expression at diencephalon and telencephalon was examined by WISH on wild type (A), *gli2a*$^{i276/i276}$ (B), wild type treated with cyclopamine (C), *gli2a*$^{i276/i276}$ treated with cyclopamine (D), and *smo*^{i640} (E).

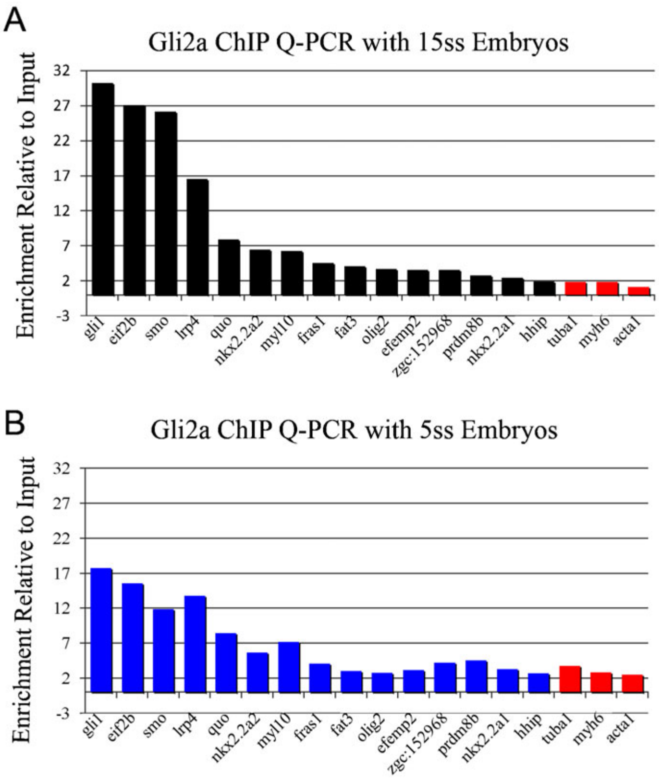

Fig. S4. Validation of ChIP-seq peaks by Q-PCR.

Biology Open

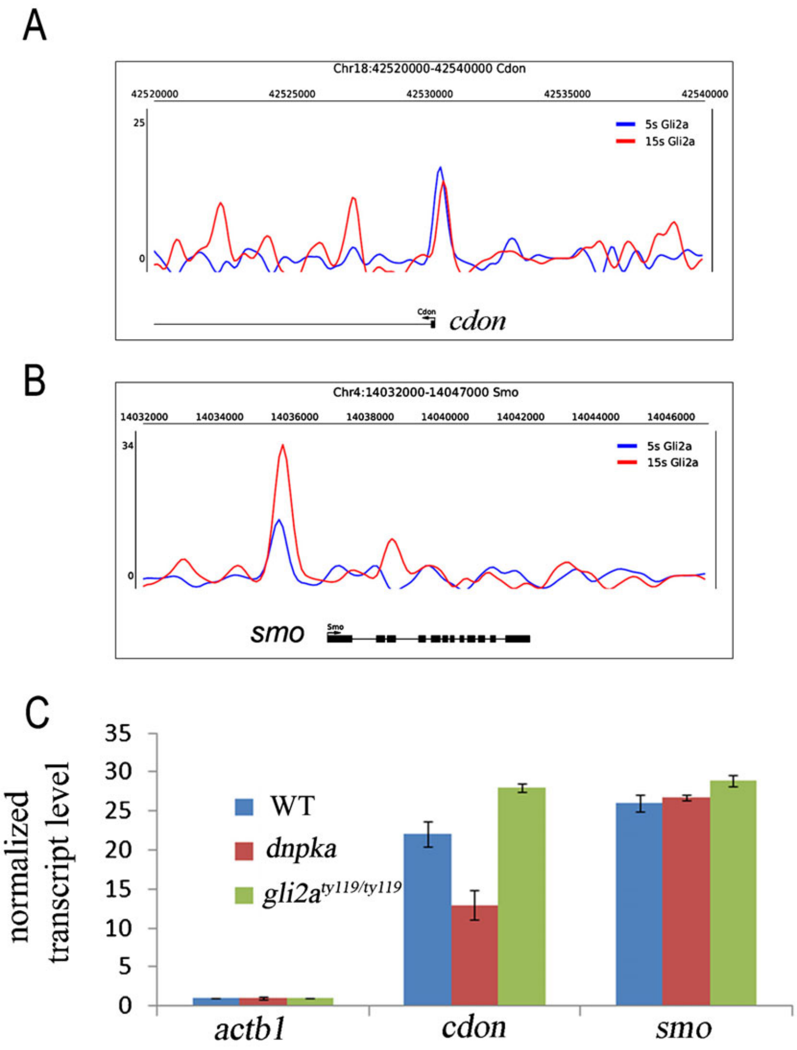

Fig. S5. Analysis of *smo* and *cdon* as putative Gli2a targets. Gli bound region on *cdon* (A) and *smo* (B), and effect of dnPKA and *gli2a*$^{ty119/ty119}$ on their transcription level (C).

Biology Open

Biology Open

Table S1. Sequences of primer pairs used for Q-PCR validation of ChIP-seq peaks.

gli1F	5′ TGGGCTGCTGCTGCTTCTT 3′
gli1R	5′ TCTGGCTTGTGATTGGCTGATA 3′
eif2bF	5′ CCACCCACAGACACTCCCAG 3′
eif2bR	5′ CACCCTCTTCCTCCGCTCTC 3′
smoF	5′ CATGGCGGCTGTAGAATGAGA 3′
smoR	5′ CTCTCCTCATTGGTGTTTGTGTG 3′
lrp4F	5′ ACAGAGCAGAGGGAAAATAAGACC 3′
lrp4R	5′ TGACGCATGGCTGCTGTCA 3′
quoF	5′ CGGTGCTGATCGAGTCTCTC 3′
quoR	5′ GCGCCTCAAACACTGAGAGAG 3′
nkx2.2a2F	5′ CCAGAGGGAGCCTTTCATTGc 3′
nkx2.2a2R	5′ TGATGTGAATGTTCAGTGGGCT 3′
myl10F	5′ GACGGAATGAAAGGCTGATAGAG 3′
myl10R	5′ TGCTCCGCTCCGTGTTTTG 3′
fras1F	5′ GGTCTGTTTCAGGCCCACAC 3′
fras1R	5′ CAAAGCAGCAGAAGTAAACAGCA 3′
fat3F	5′ GCGTCCTGTCCAGCGTCAT 3′
fat3R	5′ GCACTTTCCACAGCCACCAG 3′
olig2F	5′ TGTGATAGTCGCGGTCTGTCTG 3′
olig2R	5′ GATGTGAACCACCCTGAGCC 3′
efemp2F	5′ GACAGCTGGACTGTGAATGTATGC 3′
efemp2R	5′ GCAGACCAGGAATTTTCATCCT 3′
zgc:152968F	5′ TGCTGCACAGGAGCAAGAGG 3′
zgc:152969R	5′ ACATTCTGGCTCAAGCACACTC 3′
prdm8bF	5′ TTAATGCAATATGTATCCACGAGGT 3′
prdm8bR	5′ CAACTGCTGAGGCGGTCAA 3′
nkx2.2a1F	5′ AAAGCACATCAGGCTTATCGGT 3′
nkx2.2a1R	5′ CGTTGAGACACCGCACAGTTA 3′
hhipF	5′ TGTAAACAACCTCCGTAAATCGAC 3′
hhipR	5′ ATGCAATATGGCTTTTTGTCACA 3′
tuba1F	5′ GATGCCCATGAGAATTTAGTGCT 3′
tuba1R	5′ TCATCTTCAATGCCTCCTTCATC 3′
myh6F	5′ CAATCCTGTCGCCATTCTTTAA 3′
myh6R	5′ CTGTTCATTTATCCGCCCTCA 3′
actb1F	5′ ACCGTGCCCTTGTTTTGTATC 3′
actb1R	5′ TGTTAGATGCAACCGTATTAAGCA 3′

Table S2. Sequences of primer pairs used for Q-PCR analysis of *smo* and *cdon* expression.

smoF	5′ACCCACTGGAACACCCATTC 3′
smoR	5′CTGGCCGTACTTCCTTCCTTC 3′
cdonF	5′CTGGAGCAGCAGAGAGGAGTTC 3′
cdonR	5′TCAGGCTTCCAGTAGAGATCCA 3′
actb1F	5′GCTTTCCCCGAGAGGACAAC 3′
actb1R	5′GTCATGGACGCCCATTGTGA 3′

Table S3. gli2a mutant viability.

Parental genotypes	Number of embryos at 50% epiboly stage	Number of larvae surviving at 7 dpf	Number of fish surviving at one month	Number of homozygotes amongst 24 fish
gli2a^{i276}/+×*gli2a*^{i276}/+	80	77±3	73±2	5±1
gli2a^{i276}/+×*gli2a*^{yot}/+	80	24±3	N.T.	N.T.

N.T. = not tested.

Biology Open

Table S5. Putative Gli2a target genes with spatially restricted expression patterns.

Gene name	Expression patterns
arid1a	last three somites
atp6ap2	ear
bcl6b	head region, like krox20
bcor	head and the region between yolk entension and tailbud
cacna1c	somite, area below ear
camk2g	notochord
cdon	neuron crest
daam1b	slow muscle and notchord
dlg4	neural tube
dlgap2	somite ear
efemp2	slow and fast muscle
exoc2	neural tube
fat3	neural tube
foxq1	two anterial ventral lines
fras1	slow and fast muscle
hnmt	adaxial cells
lamc1	fin fold, notchord
lnx2b	pronephros, ear and tailbud
lrp4	somite tail bud
mpp5a	neural tube
myl10	slow muscle
olig2	neural tube
prdm8b	adaxial cells
quo	slow and fast muscle
rfx4	neural tube
sox6	fast muscle

Table S6. Comparison of GBRs associated with *ptch* genes in mouse and zebrafish.

Gene name	Peak location[1]	Conservation	Overlapping with fish Chip-seq[2]
ptch1	63910111–63911676	Mammal	
Chr13: 63520754–63574741	63575251–63577130	Mammal, fish	Peak I
	63960043–63961972	Mammal	
	63571416–63574567	Mammal, fish	Peak II
	63537116–63520754	Mammal	
	63577383–63579734	Mammal, chicken	
	63580817–63584094	Mammal, fish	
	63989338–63990236	Mammal	
	63728561–63729448	Mammal, chicken	
	63734451–63735522	Not conserved	
	63978718–63979650	Mammal	
	63533758–63535600	Mammal, chicken, fish	
	64029689–64030526	Mammal	
	63567246–63568264	Mammal	
ptch2	116595951–116597000	Mammal, fish	Peak I
Chr4: 116594287–116612762	116588169–116588714	Mammal	
	116593083–116594782	Human, dog, mice	

[1]All locations here are based on Mouse Genome Version February 2006 (NCBI36 mm8).
[2]Peak numbers are consistent with Fig. 5.
